# Graph-Driven Reaction Discovery: Progress, Challenges, and Future Opportunities

**DOI:** 10.1021/acs.jpca.2c06408

**Published:** 2022-10-03

**Authors:** Idil Ismail, Raphael Chantreau Majerus, Scott Habershon

**Affiliations:** Department of Chemistry, University of Warwick, CoventryCV4 7AL, United Kingdom

## Abstract

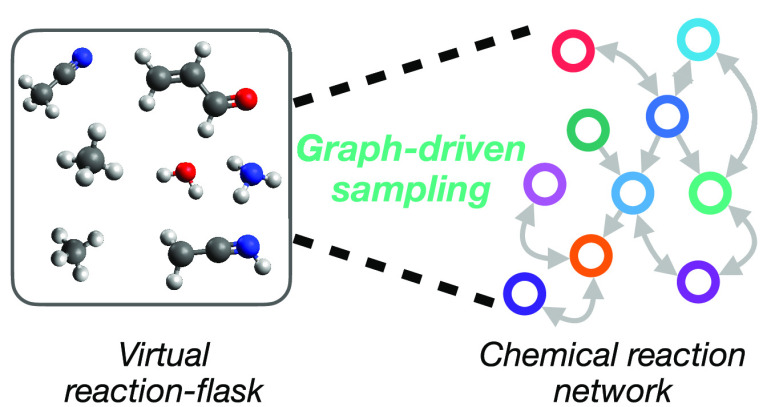

Graph-based descriptors, such as bond-order matrices and adjacency matrices, offer a simple and compact way of categorizing molecular structures; furthermore, such descriptors can be readily used to catalog chemical reactions (i.e., bond-making and -breaking). As such, a number of graph-based methodologies have been developed with the goal of automating the process of generating chemical reaction network models describing the possible mechanistic chemistry in a given set of reactant species. Here, we outline the evolution of these graph-based reaction discovery schemes, with particular emphasis on more recent methods incorporating graph-based methods with semiempirical and *ab initio* electronic structure calculations, minimum-energy path refinements, and transition state searches. Using representative examples from homogeneous catalysis and interstellar chemistry, we highlight how these schemes increasingly act as “virtual reaction vessels” for interrogating mechanistic questions. Finally, we highlight where challenges remain, including issues of chemical accuracy and calculation speeds, as well as the inherent challenge of dealing with the vast size of accessible chemical reaction space.

## Introduction

**Figure 1:**
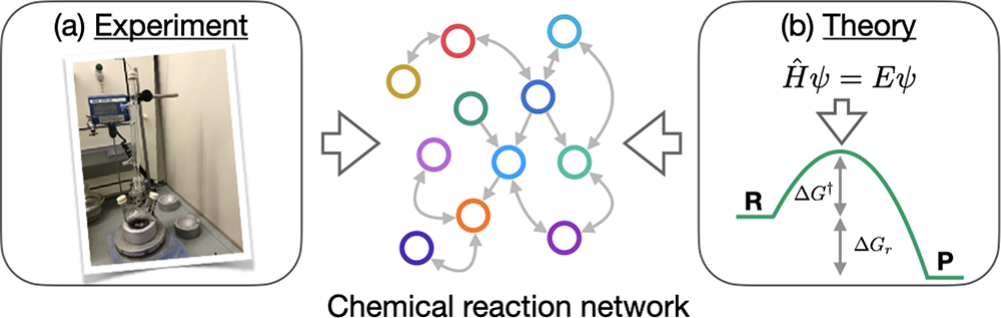
Chemical reaction networks (CRNs) serve to connect (a) experimental synthesis and characterization of reactive systems to (b) *ab initio* characterization of individual elementary reaction steps.

The concept of a chemical reaction network (CRN) provides a unifying theory linking experimental and computational studies of chemical reactivity in complex systems.^1−12^ Consider a reaction vessel in a laboratory, a catalytic reactor in an industrial plant,^13−15^ the upper atmosphere of an extra-solar planet,^16−18^ or the interface between dust grains and air in our own upper atmosphere,^19,20^ these are all environments where chemical reactions will occur and may involve large numbers of different reactive species, participating in equally large numbers of chemical reactions which may span several orders-of-magnitude in reaction rates. However, despite their apparent differences in chemistry and reactivity, all of these examples can be mapped onto a CRN describing the full collection of reactants, products, and reaction thermodynamic and kinetic parameters; as such, CRNs provide a simplifying framework enabling chemists to understand how an experimentally observed collection of chemical product species emerges from the initial soup of reactants in a reaction vessel (Figure 1).

At this point, it is useful to define a CRN; in the following discussion, a CRN is defined as a network in which the nodes (or vertices) correspond to unique chemical species and the edges correspond to the set of possible chemical reactions that interconvert the chemical species.^1,3,7−9,21−27^ Each node is typically defined by labeling the corresponding molecular species, as well as the corresponding thermodynamic properties such as Gibbs free energy.^28^ Similarly, each edge (reaction) in the CRN is defined by identifying the reactants and products of the reaction, and the kinetic characteristics (namely, the activation free energy barrier or the reaction rate).^28,29^ It is worth noting that CRNs can be defined as either (i) using nodes that identify the chemical structure of all *N* atoms in the entire reaction system or (ii) using nodes that identify the individual molecular species generated in the reaction systems. These two approaches are somewhat interchangeable, albeit demanding different “book-keeping”, and we make no particular distinction in what follows.

Importantly, the identifying characteristics of the nodes and edges in a CRN (namely, the thermodynamic and kinetic properties of all species and reactants) can, in principle, be evaluated using the machinery of *ab initio* quantum chemistry and statistical mechanics; for example, free energies of molecular species can be readily evaluated using the usual rigid-rotor/harmonic oscillator model, while reaction rates can be accessed in the first instance via transition state theory (TST).^28−31^ Importantly, these CRN characteristics enable one to perform direct kinetic simulations starting from some assumed initial concentrations of all molecular species; in other words, once a CRN is fully defined and characterized it can be used to predict the ensemble-level emergent behavior of the system, including transient species concentrations and long-time equilibrium product distributions. These same properties can, of course, typically be observed by observations of the corresponding experimental setup; in other words, the concept of the CRN, and its generation by *ab initio* quantum chemistry, provides a direct link between computation and experiment.

So, as noted above, complex chemical reaction set-ups (spanning from highly controlled, laboratory-based reaction set-ups to unobserved reactions occurring on ice grains in interstellar space)^16,17,32−37^ can in principle be mapped onto CRNs. However, now consider turning this viewpoint on its head; if we can directly generate a CRN describing and characterizing the full set of reactions and chemical species in some reactive system, then we would have an *in silico* method which is capable of *predicting* how complex chemical systems will evolve. In such cases, CRNs parametrized by *ab initio* quantum chemistry then serve as “virtual reaction vessels” which can be used to mirror and predict experimental studies of complex chemical reaction systems (Figure 1) before the potential effort and expense of real-world experimentation. However, this challenge demands new, more efficient, and more accurate simulation tools to provide automated workflows capable of delivering computational-based insights into experimental CRNs; as we describe below, this offers a wide range of emerging opportunities for computational chemists.

The generation of thermodynamic and kinetic data for all of the elementary chemical reactions in a CRN using methods based on *ab initio* quantum chemistry is itself an enormously active field of research; several relevant challenges in this area are discussed later. However, the focus of this article is instead on automatic reaction discovery (ARD)^7,9,10,12,25,27,32,38−41,41−50^ methods that can be used to “grow” a CRN (namely, the set of all reactive chemical species and the allowed chemical reactions).

**Figure 2:**
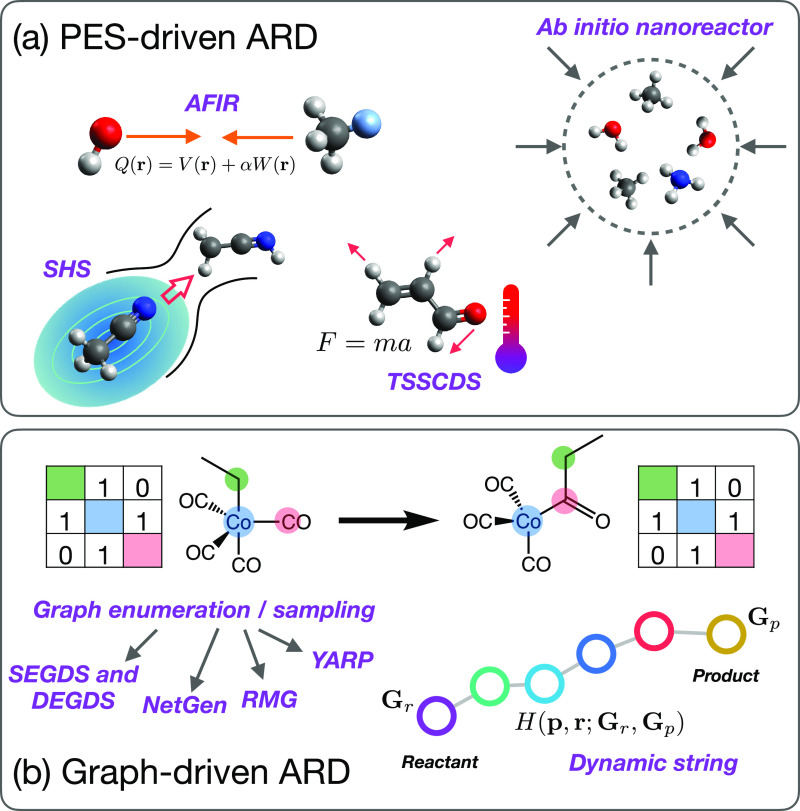
Overview of (a) PES-driven ARD schemes (e.g., AFIR, *ab initio* nanoreactor, SHS, and TSSCDS), and (b) graph-driven ARD schemes (e.g., single- and double-ended graph-driven sampling [SEGDS, DEGDS respectively], NetGen, RMG, YARP).

After emerging several decades ago, somewhat in parallel with computational methodologies for organic retrosynthesis,^11,24,51−58^ computational ARD is an increasingly useful approach to address challenges in molecular reaction design and development; a number of excellent articles have focused on ARD from the viewpoint of retrosynthesis and cheminformatics, wherein molecular structural detail is often replaced with string-based representations of molecular species and reactions.^11,24,51,53,56,59−62^ In contrast, the past decade or so has seen a rapid growth in ARD methodologies which focus on using molecular structural models, in combination with *ab initio* quantum chemistry, as the drivers for CRN generation and characterization. For the purposes of this articles, we note that such approaches broadly fall into two different categories: (i) those that employ molecular dynamics or related sampling methods to generate new molecular species and (ii) those that use discretized molecular representations, in the form of adjacency matrices or bond-order matrices (generically referred to as *graphs* here), to enable generation of CRN components (Figure 2).

Within the first class of methods, a particularly well-known example is the *ab initio* nanoreactor approach developed by Martinez and co-workers.^40,63^ Here, a simulation cell containing a set of initial molecular reactants is modeled using *ab initio* molecular dynamics, with periodic application of an artificial “piston” being used to drive molecules together in order to form new product species; mapping and tracking unique molecular species allows one to subsequently build up a picture of the sampled CRN. This approach has been applied to a wide range of different molecular systems, ranging from the classic Urey–Miller-type system (describing the emergence of complex organic molecules from simple precursors)^64^ to the decomposition of energetic materials such as nitromethane.^65^ A different approach to integrating molecular dynamics (MD) simulations and CRN generation is provided by the “transition state search using chemical dynamics simulations” (TSSCDS) method of Martínez-Núñez;^39,43−46^ here, high-energy (or high-temperature) MD simulations (employing semiempirical or *ab initio* PESs to enable correct description of electronic structure changes during chemical reactions) are initiated for a given molecular species (or collection of species), driving the making or breaking of chemical bonds. Unique species and reactions are subsequently catalogued in order to iteratively build a CRN. Other examples of ARD methods which fall into this category include the artificial force induced reaction (AFIR) method,^49^ in which molecular species are driven together during geometry optimization on a potential energy surface (PES) which incorporates an effective potential term that is increasingly biased toward molecular close contacts. The elegant simplicity of AFIR means that it has been widely applied to a range of different reaction examples, including selected steps in homogeneous catalytic reaction cycles.^66,67^ Like AFIR, the scaled hypersphere searching (SHS)^47,50^ method also introduces a distortion of the underlying PES, this time in the form of an effective force that quantifies PES anharmonicity (and hence likely low-barrier reaction routes); this approach has been fruitfully used to investigate molecular systems (such as formaldehyde, propyne, and alanine) and has also been adapted as the basis of a metadynamics sampling scheme for high-dimensional systems.^68^ More complete reviews of these methodologies can be found elsewhere.^10,12,47,48^

Our own work in this field^22,32,69−73^ and the focus of this article is exclusively on methods which fall into the second CRN generation category (Figure 2), being based on concepts of molecular graphs. As described below, graphs such as adjacency matrices offer a compact description of the chemical bonding in a collection of molecules; put simply, such graphs identify which atoms are bonded and which are not. Given this concept, chemical reactions can then be viewed as matrix operations which change the bonding graph, resulting in new bond arrangements and new chemical species; as such, CRNs can be built from sequences of bonding-graph changes to quickly enable exploration of chemical space. However, using such graph-based strategies, it is clear that the magnitude of sampled chemical reaction space can quickly become unwieldy as the size of the system grows; in the simplest case of a binary bonding graph, there are 2^*N*(*N*–1)/2^ possible bonding arrangements. So, one key difference between many graph-based CRN-generation methods is the approach taken to truncate the growth of the CRN in order to limit exploration to the region of chemical interest. In the well-known reaction mechanism generator (RMG), a set of reaction templates (describing allowed chemical reactions) are iteratively applied to a set of reactant species to generate a growing CRN; here, experimentally parametrized thermodynamic (e.g., enthalpy changes) and kinetic parameters (e.g., rate constants) are used to provide initial assessments of CRN characteristics, providing a route toward monitoring kinetic convergence of the CRN as a function of reaction set.^3,38,58,74−76^ The work of Zimmerman and co-workers offers a different strategy;^41,42^ here, allowed connectivity changes (typically user-defined given a target CRN problem) are used to generate product species for a given set of reactants, and subsequent automated *ab initio* schemes for robust MEP and TS characterization (e.g., growing-string method)^77^ are used to build up a quantum-chemical-based picture of a CRN within the region of chemical space defined by the allowed connectivity changes. As a further example, the work of Kim and co-workers^23,24,78^ employs a sequence of steps integration molecular fragmentation, formation of new bonds between different fragments using a basin-hopping scheme, followed by postscreening sorting of different molecular species and conformers. The resulting CRN can be subsequently explored and characterized using graph-based shortest-path schemes to identify plausible reaction pathways. Furthermore, the approach developed by Savoie and co-workers,^79^ and implemented in “Yet another reaction program” (YARP), is to enumerate reaction products based on graph-driven changes to connectivity, followed by fast screening based on semiempirical methods to simplify the resulting CRN and identify plausible reaction channels. Finally, we note here the recent report of the *Chemoton* software by Reiher and co-workers,^80^ which integrates connectivity-based reaction generation, conformational sampling, and novel TS-finding algorithms to provide a highly automated workflow merged with *ab initio* quantum chemistry calculations. There are clear similarities among these, and other, connectivity-based schemes, yet the continued emergence and refinement of these strategies demonstrates that there is scope for further development and optimization for addressing challenging technological problems across wider-ranging fields from combustion chemistry, chemical degradation, atmospheric chemistry, and interstellar chemistry.

Our recent research similarly employs molecular graphs to drive exploration of chemical reaction space in either a “single-ended” (known reactants only) or “double-ended” (known reactants and target product species) fashion; following earlier reports of a CRN-generation method based on Hamiltonian dynamics,^70,73^ we have subsequently expanded our approach to use generic reaction templates and to enable targeted generation of CRNs and reaction mechanisms which definitively lead to target products.^32,71,72^ In the following sections, we give a brief overview of these simulation techniques and highlight several recent applications. Based on our experiences to date, we also highlight a number of common challenges in these calculations, before concluding with some new possibilities for computationally discovered CRNs.

## Theory

In this section, we outline the key computational methods which we have employed in graph-driven ARD, including both Hamiltonian based dynamics schemes, and single- and double-ended CRN generation methods; further details are given in the references provided.

### Molecular Graphs

The key driver underlying several ARD algorithms, including our work described below, is the molecular graph defining the connectivity (i.e., bonding) of atoms in a system under investigation. For an *N*-atom system, a molecular graph is generically defined as an *N* × *N* matrix in which the off-diagonal elements *G*_*ij*_ identify the bonding characteristics between atoms *i* and *j*; the diagonal elements *G*_*ii*_ may, optionally, define characteristics of each atom (e.g., atomic number), although this is often not a requirement.

**Figure 3:**
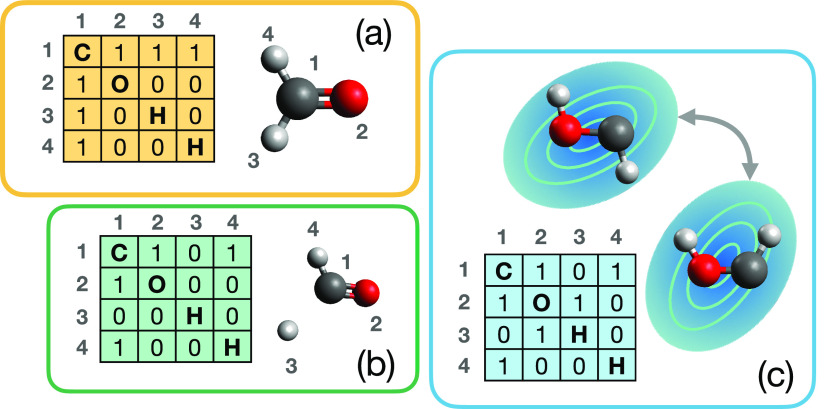
Panels (a–c) represent different regions of chemical space, naturally discretized by defining the bonding graphs shown; for example, panel (a) represents the configurational space of all systems which have the bonding graph shown (corresponding to formaldehyde). In panel (c), we note that the bonding graph shown describes both cis and trans isomers of HCOH; as such, simple bonding-graph schemes fail to distinguish conformational isomers and may require further postprocessing to account for conformational differences.

As noted above, several ARD methods have been proposed and employed that are based on the concepts of molecular graphs; depending on the exact context and method, these can be defined in different ways. For example, the method used in the YARP code^79^ is based on using bond-order matrices, where the element *G*_*ij*_ identifies the bond-order (e.g., G_*ij*_ = 1 for single-bonds, *G*_*ij*_ = 2 for double-bonds); such an approach readily enables one to keep track of atomic valences. In our work,^22,32,69−73^ we use the simpler *adjacency matrix*, defined as
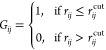
1Here, *r*_*ij*_ is the distance between atoms *i* and *j* in a given molecular structure, and *r*_*ij*_^cut^ defines a cutoff distance. As such, *G*_*ij*_ defined in eq 1 simply identifies whether or not two atoms *i*, *j* are bonded or not, as judged by a standard geometric definition. Typically, we define the cutoff distance as *r*_*ij*_^cut^ = α(*R*_*i*_ + *R*_*j*_), where *R*_*i*,*j*_ are the covalent radii of the respective atoms and α is a parameter to build in some flexibility in accounting for bond lengths in different molecular systems (typically α ≃ 1.1–1.2). Examples of such graphs, calculated for the H_2_CO system, are shown in Figure 3, where the discretization of chemical space by graphs is emphasized.

Molecular graphs of the form given in eq 1 offer a number of computational advantages in forming the basis of ARD schemes. For example, such matrices offer a straightforward discretization of chemical space: Differently bonded chemical species correspond to different bonding graphs, enabling simple comparison and enumeration of different molecules in a “virtual reactor”. From a computational viewpoint, graphs have the additional advantage of generally being sparse (with large numbers of zero matrix elements) and easy to manipulate or interrogate using well-developed graph processing algorithms.^81^

In the ARD algorithms developed in our recent work, we use the discrete space provided by molecular graphs to drive exploration of chemical reactions and hence generate CRNs. Specifically, we typically begin with an input set of reactant molecules (which depend on the problem at hand), defining an initial bonding graph **G**; subsequently, we generate sequences of reactive events (defined via reaction classes as defined below) in order to modify the chemical bonding (and hence graph **G**) in the system. This generation of new graphs can be done in several different modes, typically single-ended generation (in which only the initial reactants are defined^70,73^) or double-ended generation (in which both reactants and target products are defined).^32,71,72^ For all of the new graphs generated, we can obtain corresponding atomic coordinates using a “back transformation” enabled by an artificial PES referred to as a graph-restraining potential (GRP), as described below.

Before discussing the details of our ARD approaches, it is worth noting that methodologies based on graphs have associated disadvantages too. Perhaps most importantly, graphs reduce a 3D molecular structure to a discretized 2D representation; as such, all information about molecular conformation is lost. This is highlighted in Figure 3c, where the cis and trans conformers of HCOH are shown as corresponding to different regions of conformational space, but the same region of the discretized graph space. For simple molecular reactants, or systems in which reactive molecules are generally rigid (such as many organometallic complexes), the limited conformational flexibility means that this simplifying approximation can often be overlooked. For more complex molecular reactive species, conformational flexibility will inevitably force the user to decide how to treat this. In this regard, the two main approaches can be described as either (i) using sampling strategies such as MD or Monte Carlo, in combination with standard empirical force-fields (where applicable) to generate all *unique* and *relevant* molecular conformations, and include each conformer as a separate node in the generated CRN or (ii) using a *single* conformer (typically a local minimum on the PES or the globally minimal conformation on the PES where available) as a representative example of a given molecular reactant. The former approach is obviously more computationally demanding (requiring generation of possibly large numbers of conformers and assessment of their relative energetics), whereas the latter can potentially introduce uncertainty in the accuracy of calculated thermodynamic and kinetic features in the CRN (by ignoring the thermodynamic and kinetic role of interconversion between different conformers or conformer-specific reactivity). As discussed below, the challenge of conformational flexibility remains an unsolved problem; it seems likely that the solution would be strongly system-specific.

### Reaction Classes and Valence Constraints

**Figure 4:**
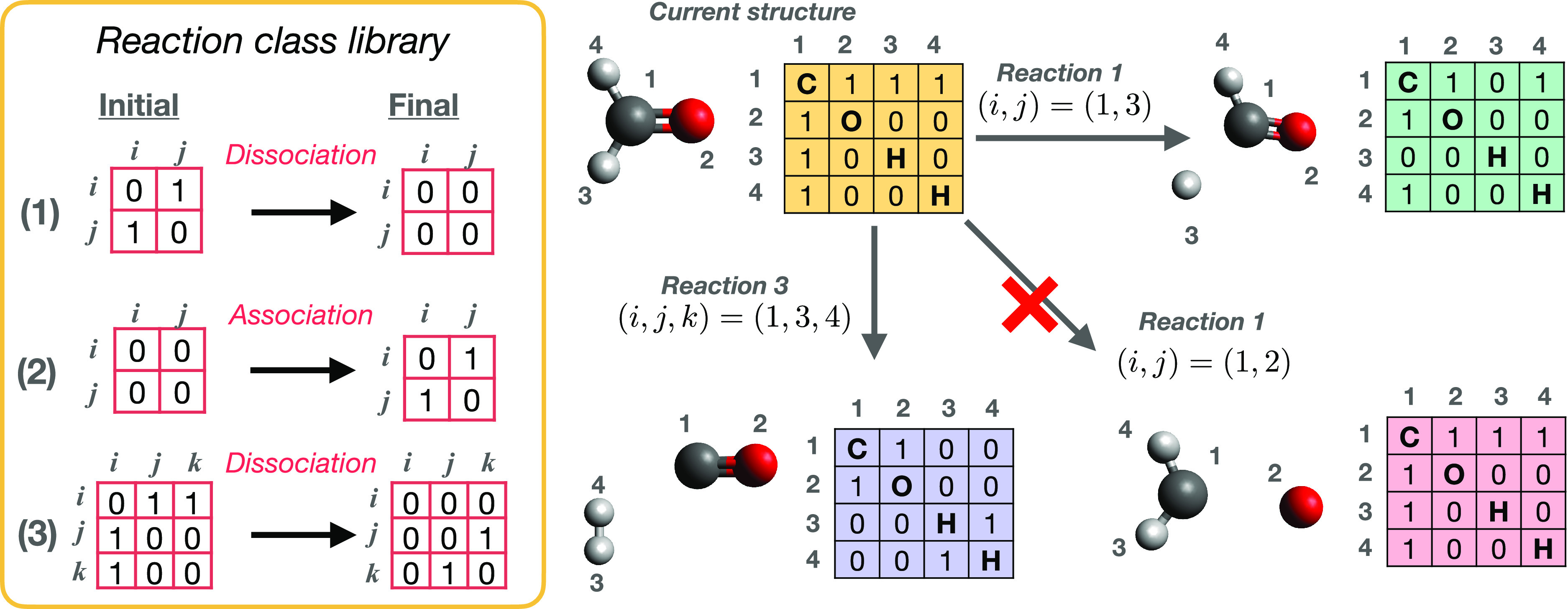
Overview of reaction class definitions, as employed in our recent work.^22,32,69−73^ The left-hand panel shows the initial and final bonding graphs for three representative two- or three-atom reactions, namely, (1) dissociation, (2) association, and (3) diatomic dissociation. Here, the bonding matrices show the connectivity for atoms (*i*, *j*) or (*i*, *j*, *k*) before and after a given reaction is applied to a reactant set; by selecting a reaction class and related atomic indices, one can automatically induce reactions on a system’s bonding graph to generate new products. The right-hand side shows illustrative examples of this scheme. Starting from formaldehyde, application of reaction class (1) to atoms (*i*, *j*) = (1, 3) results in a valid product structure (assuming that the allowed valence range of hydrogen includes zero). Similarly, applying reaction class (3) to atoms (*i*, *j*, *k*) = (1, 3, 4) results in dissociation of molecular hydrogen, which is again considered here to be a valid structure. However, applying reaction class (1) to (*i*, *j*) = (1, 2) would here be rejected as a valid reaction, assuming that the allowed valence range of oxygen does not include zero. These examples illustrate how application of generic reaction classes, combined with standard valence constraints, can be used to quickly build a CRN.

As well as defining the connectivity of a given molecular structure, graphs can also quite straightforwardly be employed to define reaction classes (also known as reaction templates or reaction families)^57−59,70^ and their operation on a given molecular structure. In our approach, in common with previously demonstrated schemes noted above, we adopt the strategy of defining an *allowed* set of chemical reactions by defining the corresponding bond-change matrices associated with each reaction class (Figure 4). We note that these reaction classes can be defined quite generically; for example, as shown in Figure 4, atomic association/dissociation reactions can be defined by 2 × 2 matrices describing the connectivity before and after the reaction. Similarly, diatomic dissociation reactions can be defined using 3 × 3 generic matrices, again describing bonding before and after (alternatively, one can define a single “bond-order change” matrix by defining the product and reactant reaction class graphs). In such a way, these generic reaction classes can be applied to any set of atoms that match the target pattern in the initial bonding matrix. For the case of two-atom reactions, it is clear that only two possibilities exist here (i.e., association and dissociation): For three- and four-atom reactions, the size of the reaction class library will obviously grow; however, it is straightforward to introduce “chemical knowledge” into the reaction class library, for example, by removing selected reaction classes that might be expected to have a low probability for a given chemical system.

Once reaction classes are defined, these reactions can then be “applied” to a given reactant structure, resulting in the generation of new product species with altered bonding matrices. As noted above, this approach forms the basis of a number of successful computational strategies for autogenerating CRNs and for discovering new reactions; the different schemes that are based on graphs differ primarily in either the approach taken to generate CRNs (for example, using either complete enumeration of all reaction outcomes^79^ or focusing on more defined regions of reaction space),^70,73^ as well as the different strategies used to characterize CRNs (for example, using parametrized thermodynamics^3,38,58,74−76^ or *ab initio* energy calculations).^41,42,77^ Again, details for the various graph-driven strategies can be found in the original reports and recent excellent reviews.^10,12,47,48^

However, a common theme, and one of the useful strengths associated with a graph-based strategy, is the ability to quickly identify “nonchemical” reaction products that exhibit nonphysical chemical bonding patterns. An example of this is shown in Figure 4; assuming that we had judged that the dissociation of a lone oxygen atom from formaldehyde to be a very unlikely event (for example, based on chemical intuition or previous knowledge of bond energetics), then any reaction that results in an oxygen coordination number of “zero” can be immediately discounted during the ARD calculation. This “reaction rejection” can be readily achieved using the bonding matrix of the product system, without having to perform *ab initio* energy evaluations or other expensive assessments. Finally, it is worth noting that the discretization of chemical reaction space provided by bonding matrices also enables other rejection schemes to be readily incorporated; for example, it is straightforward to focus on generating reactions which only involve a defined subset of “active” atoms in a given reactant, a feature which is more challenging to achieve using MD-based schemes.

### Graph-Driven Sampling Schemes

We now highlight how, based on the concepts of bonding graphs and reaction classes, we have in the past few years explored several different computational schemes which are aimed not at explicit enumeration of all molecular components in a CRN, but rather toward generating more tailored mechanistic hypotheses for specific reactive (or catalytic) systems.

#### Dynamic Strings

In our initial work,^70,71,73^ we showed how ARD can be mapped onto the molecular dynamics of a “dynamic reaction string” connecting configurations that are restrained to regions of chemical space defined by endpoint bonding graphs. Here, we define an effective Hamiltonian that describes the kinetic energy and PES of such a restrained reaction path, as follows:
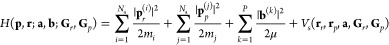
2This classical Hamiltonian describes a reaction string comprising a total *M* = *P* + 2 configurations in the (3*N*_a_ – 6)-dimensional space of a given reactive system containing *N*_a_ atoms. The two endpoints of the string (**r**_*r*_, **r**_*p*_) are connected by a series of *P* intermediate configurations, in a similar manner as in the familiar NEB method.^82−87^ In our work, rather than defining the *P* intermediate configurations in the (3*N*_a_ – 6) Cartesian coordinates of the system, we instead chose to use a set of Fourier coefficients **a** that are themselves associated with a set of conjugate momenta **b**; our reason for this choice at the time was based on seeking improved stability of time-evolution in the intermediate images.^70,73^ The endpoints also have associated momenta (**p**_*r*_, **p**_*p*_); as such, the first three terms in eq 2 represent the *total* classical kinetic energy of the endpoints and the intermediate Fourier coefficients (which are associated with a fictitious mass μ).

The PES associated with eq 2 is
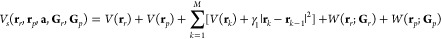
3Here, the first two terms are the PES values at the string endpoints; the third term is the corresponding PES values at the intermediate images, in addition to a NEB-like spring term that helps avoid “kinks” along the reaction path. The positions of the intermediate images are derived from the endpoint coordinates and the set of Fourier coefficients **a**.^70,73^

The final two terms in eq 3, *W*(**r**_*r*_; **G**_*r*_) and *W*(**r**_*p*_; **G**_*p*_), define GRP functions. This empirical function lies at the heart of several of our recent studies; in short, this PES term *W*(**r**; **G**) is designed to be a minimum only if the configuration **r** is definitively consistent with the given bonding graph **G**. If the bonding matrix associated with the current configuration **r** does not match that in **G**, then *W*(**r**; **G**) provides a force that drives **r** toward regions of space in which the bonding matrix evaluated from any configuration matches the target bonding matrix **G**.

With the definition of the classical Hamiltonian of eq 2, it is possible to derive equations-of-motion describing the time evolution of the reaction endpoints and the intermediate images (or Fourier coefficients). The PES of eq 3 ensures that the endpoints are restrained by the GRP to regions of configuration space that are consistent with the bonding graphs **G**_*r*_ and **G**_*p*_, whereas the intermediate images will roughly approximate an MEP connecting the two endpoints. As such, eq 2 does not inherently sample chemical reactions; for a given fixed pair of endpoint graphs (**G**_*r*_, **G**_*p*_), the Hamiltonian enables sampling of the configuration space of a reaction string connecting endpoint configurations consistent with the defined graphs. So, to enable sampling of chemical reactions *and* MEP-like reaction strings, we use a series of periodic “hops” in the graph space; here, one of the predefined reaction classes described above is selected, as well as a corresponding set of reactive atoms, and one of the endpoint graphs is then updated to reflect this change. This change in endpoint graph must be consistent with atomic valence constraints, as described above. From eq 2, it is clear that forces from the GRP terms act to push the configuration into the region of space consistent with the new bonding graph. This cycle of MD sampling and periodic endpoint graph changes is repeated for a range of different initial molecular reactants, for example, incorporating new species generated in earlier trajectories. The result of this simulation approach is generation of a CRN describing the sampled molecular species, as well as good initial guesses for the MEP connecting different reactants and products (i.e., snapshots of the reaction string). This information can then be used in standard schemes to evaluate thermodynamic and kinetic properties for all generated reactions.

This MD-based approach is naturally quite computationally demanding, requiring one to perform string-based MD simulations on PESs which enable treatment of bond-making and -breaking processes.^70,73^ To address this challenge, we have previously employed fast semiempirical or parametrized PESs in these MD simulations, for example, using density function tight-binding (DFTB)^88^ or the ReaxFF force-field.^89^

#### Single- and Double-Ended Graph-Driven Sampling

The Hamiltonian-based system described above enables one to generate and characterize quite complex CRNs; as shown below, applications to systems such as homogeneous catalysis by organometallic complexes illustrates the potential of this strategy. However, the requirement to perform many PES evaluations at each MD time step (i.e., for the *M* images in the reaction string) places significant computational demands on this strategy, even using faster semiempirical methods.^71^ In addition, depending on how frequently graph moves are performed, the reaction string system can spend a considerable amount of time simply sampling configuration space, rather than performing the chemical reaction space sampling required for CRN generation.

**Figure 5:**
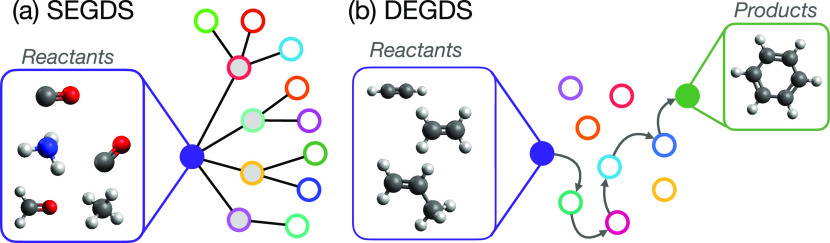
Comparison of (a) SEGDS and (b) DEGDS. In the single-ended scheme, repeated application of reaction classes to different sets of reactive atomic indices generates a large number of different structures (shown here as circular nodes) connected through elementary reaction steps (shown here as connections); characterization of each generated reaction using, for example, *ab initio* quantum chemistry or AI/ML, ultimately enables chemical insight. In the double-ended scheme, plausible mechanisms are generated that definitively connect input reactants to a target product; repeated generation and characterization of different mechanisms enables one to home in the “most likely” reaction mechanism based on thermodynamic and/or kinetic grounds.

To address this computational expense, our recent work has taken the approach of replacing the reaction string with a single representative configuration defined by a reactant graph **G**_*r*_;^22,32,72^ starting from this configuration, repeated application of randomly selected reaction classes on randomly selected subsets of atoms (subject to user-defined atomic valence constraints) generates sequences of bonding graphs which represent the chemically accessible space of the system. For each new graph that is generated, a corresponding set of atomic Cartesian coordinates can be generated by performing optimization under the action of the GRP, starting from the previous system configuration; this approximate new structure can be subsequently optimized (e.g., using semiempirical methods) to generate accurate molecular structures. Finally, unique reactant/products pairs can be cataloged to build up a CRN, and the initial/final configurations generated at each graph move step can be used as endpoint configurations for further MEP and TS-finding calculations. This general strategy, of iteratively generating new products using walks in chemical graph space, is common to a number of different graph-based strategies, with differences typically observed in the definitions of the relevant graphs, generation of real-space configurations, and imposition of molecular valence and bonding constraints. In the following, we will refer to this strategy as single-ended graph-driven search (SEGDS; Figure 5).

However, while variants of SEGDS-type schemes have been demonstrated previously,^3,7,10,23,24,38,41,42,58,74−76,78,79^ an important underlying challenge in such methods is the sheer scale of the chemical search space (as also discussed later). For even a moderately complex chemical system, containing a few tens of atoms, the number of possible unique reaction products grows dramatically with system size, even if one employs typical valence-constraint schemes to remove chemically irrelevant molecules. Employing bond-based schemes to focus attention on reactions involving “few-bond” changes (for example, reactions that only break/form a maximum of two bonds in a given elementary step)^79^ further helps limit the chemical reaction space, but the fact remains that complete enumeration of all relevant reactions in a complex system might often be beyond the scope of current computational power.

However, it is worth noting that a common goal in ARD simulation studies is often not to generate or fully enumerate an entire CRN but instead to seek out mechanisms which lead from well-defined reactants to target products. To answer such mechanistic questions, which are common in important fields ranging from organic synthesis to organometallic catalysis, single-ended approaches may not be necessary to answer the question at hand; instead, a more focused approach is required for generating “mechanistic hypotheses” given target reactants and products.

To deliver this goal, we have modified SEGDS to create a double-ended graph-driven sampling (DEGDS) algorithm; the aim here is to take as input a set of reactant species *and* a target product molecule, as well as to identify entire mechanisms (i.e., sequences of reaction classes and reactive atoms) which connect these structural endpoints.^32,72^ The key to DEGDS is to cast the problem of mechanism proposal as an optimization problem that can be readily addressed using standard discrete optimization strategies. First, following the discussion of reaction classes and reactive atoms in section, we note that a mechanism can be straightforwardly encoded as a sequence of integers that defines (i) the reaction class at each step in a mechanism and (ii) the indices of those atoms that participate in each reaction step. As illustrated in Figure 5, starting from some input reactants (with bonding graph **G**_*i*_), the result of operating with such a reactivity sequence is a new graph  given by
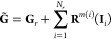
4Here, *N*_r_ elementary steps is the maximum allowed number of elementary steps in a proposed reaction mechanism and *m*(*i*) labels the elementary reaction class occurring at reaction step *i*. **I**_*i*_ labels the set of atomic indices participating in reaction step *i*. Finally, **R**^*m*(*i*)^ denotes the graph operation performed by reaction class *m*(*i*), as illustrated in Figure 4. This sequence of reaction classes and reactive-atom indices provides a convenient discrete space defining a mechanism.

In order to seek out mechanisms that definitively lead to formation of a known target product, we define an optimization function *F* that is constructed such that it is exactly zero when a molecule in the graph generated by the operation of the current reaction/atom sequence matches the target product molecule; as such, to identify the set of reactions and associated atoms that lead from reactants to products, we simply perform optimization of the integer sequence comprising the set of reaction classes *m*(*i*) and reactive atomic indices **I**(*i*), using *F* as our cost function. This can be achieved using any number of different optimization algorithms; to date, we have exclusively used simulated annealing (SA).^32,72^

The definition of *F* is somewhat flexible, and to date we have used two different approaches. In our initial simulations using DEGDS, we simply defined *F* using element-wise comparison between the bonding graphs for the target product and that produced by applying the sequence of current reaction classes and reactive indices:
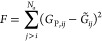
5This simple least-squares function will obviously be zero when every bonding-matrix element in  matches that in the target product graph **G**_p_. However, this cost-function has important disadvantages, particularly for complex reaction systems. In particular, it requires one to unambiguously define the target bonding pattern of *all* molecules in the target product system, and it also neglects to account for atomic permutational invariance. For example, in complex many-molecule systems such as the interstellar reactions considered below,^32^ defining the bonding pattern of all molecules in the product set is not relevant; one might only interested in formation of a particular given species, without particular regard to the rest of the product species. Furthermore, for complex reactive systems, there will be a large number of different mechanistic possibilities that might form a given target product; for example, in the interstellar reactions discussed below, there are different routes to form benzene using different carbon and hydrogen atoms from different reactant species. However, the cost function of eq 5 does not correctly reflect this permutational symmetry and instead requires that the *indices* of atoms comprising the final product are known in advance; mechanistic proposals that form the correct target product, but from a different set of index-labeled atoms from the input target product, would be flagged as having *F* > 0 (and hence not identified as plausible mechanisms) because eq 5 does not respect the fact that a given target product could potentially be formed from different subsets of reactant atoms and molecules.

To address these issues, we have recently modified our initial strategy to employ optimization functions of the form:
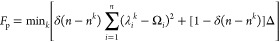
6Here, when calculating the optimization function *F*_p_ for a given product bonding-graph , we scan over all product molecules *k* in the system (as can be readily identified from the bonding graph itself). The first term in eq 6 is only evaluated when molecule *k* has the same number of atoms (*n*^*k*^) as that in the target product structure (*n*). When this condition is met, the “effective distance” between molecule *k* and the target product molecule is given as the sum of squared-differences between the eigenvalues of the mass-weighted bonding graphs. When the number of atoms in *k* is not the same as that in the target product, we simply assign a large value *F*_p_ = Δ*a*s a penalty term. The final value of *F*_p_ is the minimum value of the calculated terms among the set of molecules in . This cost function has the significant advantage that it places emphasis on seeking out mechanisms that form a single user-defined product structure, without regard to the remainder of the reaction system. In addition, *F*_p_ is permutationally invariant to atomic indices, such that the target product can be formed by any combination of atoms (as long as they have the correct desired atomic masses).

Once a mechanism with *F* = 0 (or *F*_p_ = 0) has been obtained, atomic coordinates for all intermediate structures can be generated using the GRP, as employed previously in our Hamiltonian-based scheme. With atomistic models of all reaction intermediates in hand, further quantum-chemistry-based analyses can be performed, such as evaluating the energy changes and activation energies at each reaction step. By comparing these physical quantities across a large number of proposed mechanisms, different mechanistic proposals can be identified as being more or less likely; furthermore, DEGDS can also be used to generate a CRN in the same way as SEGDS, although the resulting CRN structure would be naturally biased toward the region of chemical space that contains structures along the paths to the target product. Finally, we note that it is, in principle, possible to modify the cost function *F* to account for the thermodynamic and kinetic characteristics of different mechanisms, driving the search for “more plausible” mechanisms rather than ranking proposed mechanisms in a postprocessing step. This alternative approach requires fast methods of assessing the energies and activation energies of different intermediates and reactions, respectively, for which AI/ML methods described below may prove useful; work in this direction is ongoing.

## Applications

Here, we highlight several recent applications of the methods above to ARD; emphasizing our interest in reaction mechanisms and chemical dynamics, we describe applications of the dynamic string and DEGDS methods to complex reactive systems, before highlighting ongoing work on multicomponent reactions and protein folding mechanisms.

### Catalysis

A key target for ARD simulations is the analysis of catalytic reactions in homogeneous or heterogeneous systems; given the enormous importance of catalysis across both academic and industrial chemistry settings, this emphasis is no surprise. As such, a number of ARD studies have investigated CRNs for catalytic species, both homogeneous and heterogeneous;^3,12,15,22,43,67,70,71,90−99^ as described below, the rise of AI/ML techniques in catalyst analysis, coupled to ARD, is a growing area.^90,94,97,100−102^

**Figure 6:**
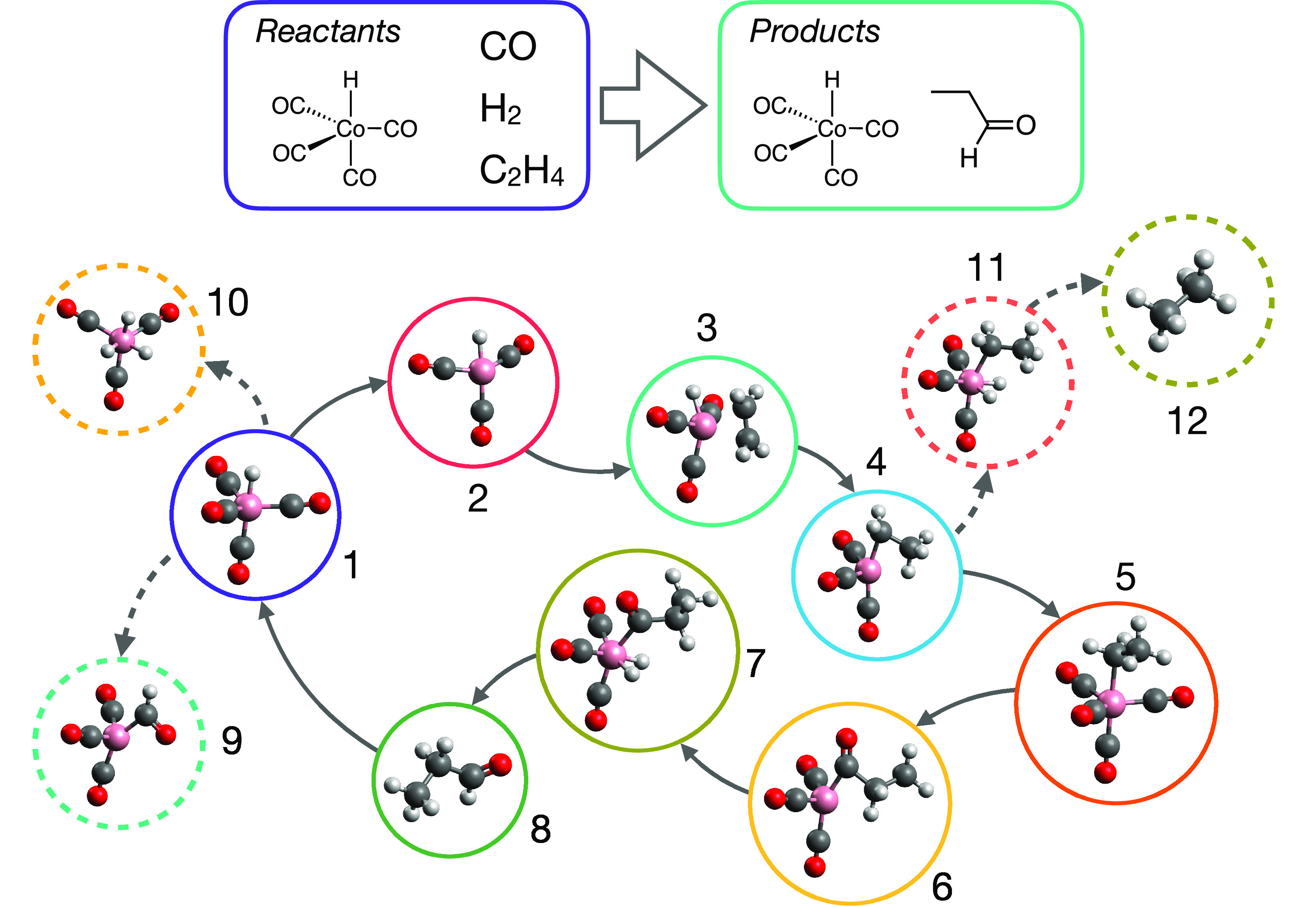
Outline of graph-based ARD study of cobalt-catalyzed hydroformylation of C_2_H_4_. The assumed reactants are shown at the top, alongside the expected products. ARD simulations based on our proposed dynamic string method^73^ generated 32 different molecular structures contained within the catalytic cycle; some of the most relevant structures are shown here, labeled 1–12. Those structures shown in solid circles (1–8) are the key intermediates and products of the expected Heck–Breslow reaction mechanism, whereas representative side products (9–12) are shown in dashed-line circles.

The hydroformylation of small alkenes, such as ethene and propene, by cobalt carbonyl complexes has served as a useful benchmark system for several different computational ARD schemes,^23,43,66,103^ including our own work.^71,73^ In this reaction (Figure 6), HCo(CO)_4_ serves as a catalysis for hydroformylation of alkenes into aldehydes; for example, a number of studies have focused on conversion of ethene into propaldehyde. This reaction follows the well-known Heck–Breslow mechanism,^104^ in which HCo(CO)_4_ loses a CO ligand to become HCo(CO)_3_; the ethene subsequently coordinates to Co and inserts into the Co–H bond. Addition and insertion of CO, following by coordination and dissociation of molecular hydrogen, then leads to elimination of the aldehyde product and the regeneration of HCo(CO)_3_. From the computational point-of-view, this reaction is quite useful to study. The mechanism is well-studied,^103,105,106^ and an experimental reaction rate law is known,^103,107^ enabling direct verification through simulations of the constructed CRN. Furthermore, the system is small enough that DFT calculations can be readily performed for elementary reactions steps, while at the same time semiempirical methods can be used to somewhat reliably enable PES exploration during application of ARD schemes.

Our initial investigation of this system,^73^ used the “dynamic string” approach described above to generate a CRN describing the hydroformylation of ethene by HCo(CO)_4_ (Figure 6). Here, we employed DFTB as the underlying PES for our string calculations, generating an initial CRN comprising 31 unique chemical structures connected by 32 chemical reactions. For all reactions, the reactants and products were geometry-optimized by DFT; subsequently, Hessian matrices were calculated, enabling evaluation of relative free energies within the standard rigid-rotor/harmonic-oscillator partition function approximation. Furthermore, for each reaction in the generated CRN, a representative snapshot of the dynamics string was used as a starting point for MEP refinement using NEB;^82−87^ following this, the TS for each reaction was optimized and characterized, enabling evaluation of activation energies and corresponding rates (using TST).^28−31^

As shown in Figure 6, the resulting hydroformylation CRN contains all of the structural intermediates and elementary reactions one might expect to see based on the Heck–Breslow mechanism. In particular, structures 1–8 in Figure 6 represent the key intermediates in the Heck–Breslow mechanism, forming aldehyde product 8 (we note that “spectator” molecules in each structure are not illustrated for clarity). However, it is also worth noting that a number of side reactions are also generated during the course of our dynamic string simulations, such as structure 9 (formed by insertion of CO into the Co–H bond of HCo(CO)_4_) and structure 10 (formed by addition of H_2_ to HCo(CO)_3_); furthermore, we note that other possible products, such as ethane (structure 12), are also generated within the CRN, highlighting the possibility to explore “off-path” reactions in catalytic cycles.

Following complete characterization of all molecular structures and reaction paths, we subsequently performed microkinetics simulations using Gillespie’s stochastic simulation algorithm (SSA).^108−110^ These simulations, performed under experimentally realistic conditions of species concentration and temperature, enabled evaluation of the rate of formation of the product aldehyde species; performing a series of independent SSA simulations for a series of different concentrations of each reactant species (i.e., HCo(CO)_4_, CO, H_2_ and C_2_H_4_) enabled identification of the overall rate law for the catalytic cycle. Our calculated rate law was broadly in line with previous experimental observations^111^ and theoretical predictions,^103^ for example, demonstrating and inverse dependence on the square of the concentration of carbon monoxide (and we note that these kinetics simulation results were later further refined by Martínez-Núñez).^43^ Furthermore, the microkinetics simulations were also extremely useful in providing much clearer insight into the mechanism, principally by enabling calculation of the reactive flux through each reaction in the CRN; such simulations were also used to identify a “minimal” CRN that was found to contain the expected Heck–Breslow catalytic cycle. Overall, therefore, these results serve as a clear demonstration of the power of ARD simulations in linking the worlds of quantum-chemical calculations to experimentally observable kinetics.

As a final comment, we note that we have subsequently investigated the same hydroformylation reaction using DEGDS.^71^ As noted above, DEGDS seeks a reaction path connecting reactants (in this case, the catalyst plus reactants H_2_, CO and C_2_H_4_) and products (in this case, the reconstituted catalyst and the aldehyde product). In our simulations, we generated 47 candidate reaction mechanisms and subsequently screened them using DFTB calculations; here, we evaluated two different descriptors quantifying the “roughness” of the reaction energy landscape for every mechanism, enabling a rough ranking of different proposals on the assumption that avoiding formation of intermediates with large energetic change relative to the previous step is desirable in catalytic processes. Closer investigation of the fewest “best ranked” mechanisms (for example, using NEB calculations for each reaction step in the proposed mechanism to identify approximate activation energies) then revealed that the expected Heck–Breslow mechanism was indeed identified in the set of “best” candidate mechanisms; these results therefore demonstrate how a combination of ARD simulations and semiempirical energy evaluations can allow fast initial assessment of candidate reaction mechanisms in complex systems, and current work is ongoing to exploit this strategy in broader catalyst design studies.

### Interstellar Chemistry

As a further application of our DEGDS scheme for mechanistic proposal, we have recently studied the formation of benzene in the interstellar medium.^32^ The formation of complex organic molecules (COMs) in the interstellar medium and in planetary atmospheres is a diverse and rapidly expanding field of interest that draws heavily on understanding the chemistry of organic radicals, neutral molecules, and ions;^2,16,33−35,37^ in addition, reactions on surfaces are increasingly studied as sources of COMs in interstellar dust clouds.^17^ The formation of benzene and higher polycyclic aromatic hydrocarbons (PAHs) is particularly interesting, given the challenges in understanding the origins of such complex species and the potential for more broadly understanding the emergence of complex chemistry in hostile environments.^16,35,37^

In our DEGDS-based ARD study, we focused on investigating the formation of benzene (C_6_H_6_) from a broad variety of smaller precursor molecules such as C_2_H, C_2_H_2_, C_4_H_3_, and C_4_H_6_. The formation of benzene in the interstellar medium has been previously postulated to result from different pathways, including ion–molecule reactions and barrierless radical reactions.^16,18,33,112−114^ As such, this system, with its diverse set of possible reactants and reaction mechanisms, serves as another useful tool to investigate the performance of ARD methods.

In our DEGDS simulations of benzene formation, we generated 2230 different candidate reaction mechanisms forming benzene; these simulations used different sets of initial small-molecule reactants, with the largest systems studied containing 96 atoms and eight different reactant species. As in our DEGDS study of hydroformylation, we prescreened the plausibility of all mechanisms by calculating descriptors quantifying the energetic “roughness” of each reaction mechanism. In addition, given the supposed low-temperature environments (∼10 K) in which the relevant benzene formation paths occur in the interstellar medium, we also focused our attention on “barrierless” mechanisms by ignoring reaction mechanisms with high-energy intermediates relative to reactants. The ultimate outcome of this screening process was the identification of around 12 unique mechanisms that formed benzene from different sets of reactants. The elementary reaction steps in each proposed mechanism were then subject to NEB MEP refinements and TS identification at the DFT level.

**Figure 7:**
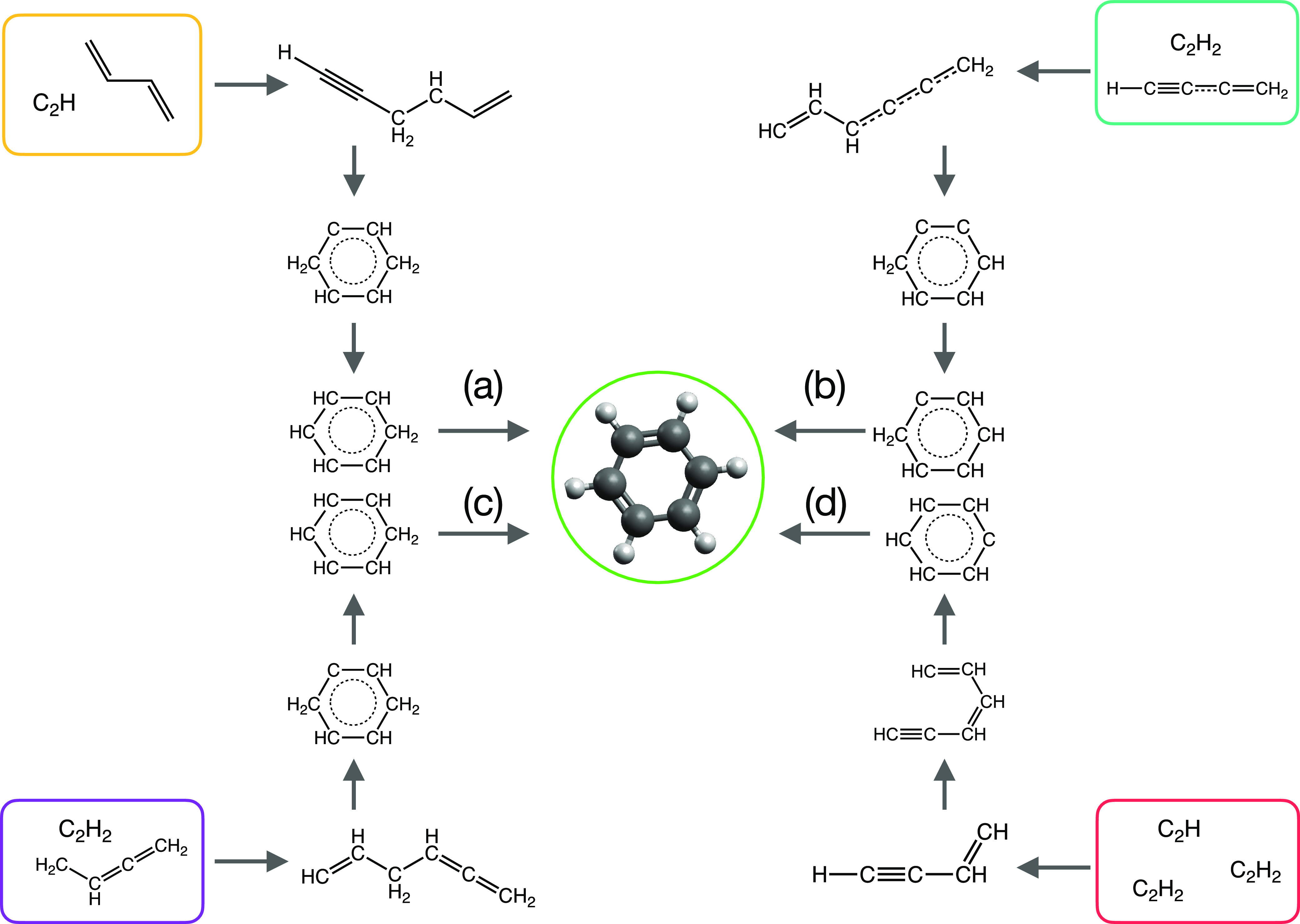
Four representative reaction mechanisms forming benzene from different initial reactant species, as identified in DEGDS simulations.^32^ Mechanism (a), discovered by our ARD simulations, corresponds to that previously identified based on experimental data.^16^

Figure 7 illustrates four of the key mechanisms identified from this ARD/screening strategy. Importantly, we did indeed identify a barrierless reaction mechanism that formed benzene by addition of C_2_H to *trans*-1,3-butadiene, followed by ring-closure, hydrogen transfer and hydrogen dissociation (Figure 7a); this is the same mechanism of benzene formation that had previously been postulated^16^ based on experiments. Further reactions (Figure 7b,c) were, after NEB refinement, ultimately found to have small barriers to initial addition of C_2_H_2_, precluding their further consideration as candidate reaction mechanisms in very-low-temperature environments. Finally, we note that mechanism Figure 7d, forming benzene from addition of C_2_H and C_2_H_2_, was ultimately found to be barrierless *and* to have lower activation energies than the proposed mechanism Figure 7a. However, we also noted that the C_4_H_3_ species formed by initial addition of C_2_H and C_2_H_2_ is in fact known to undergo a 1,2-hydrogen shift reaction to form *iso*-C_4_H_3_; this lower-barrier side reaction was not captured by our DEGDS simulation (which focuses on definitive formation mechanisms, rather than broader CRN scanning). As such, this result suggests that further work is required to both simultaneously postulate reaction mechanisms while also accounting for other plausible side-reactions; a combination of both single- and double-ended ARD schemes seems like a sensible compromise here. Nevertheless, the screening and identification of the accepted mechanism of benzene formation in these simulations is highly promising of the potential power of these simulations, especially given the complexity of the studied input reactant sets. As a final comment, it is worth emphasizing that these simulations focused exclusively on *neutral–neutral* molecular reactions, whereas reactions involving charged species are also likely to play an important role in the ISM; modifying DEGDS simulations to account for charged species could be achieved by different routes, for example, by explicitly accounting for different charge states of molecular intermediates in *ab initio* calculations or by introducing charge information into the mechanism search itself; these are ongoing projects.

### New Applications: Multicomponent Reactions and Protein Folding

Finally in this section, we highlight two ongoing projects deploying ARD (primarily using DEGDS) to generate candidate mechanisms for complex systems; we anticipate reporting results of these simulations soon.

**Figure 8:**
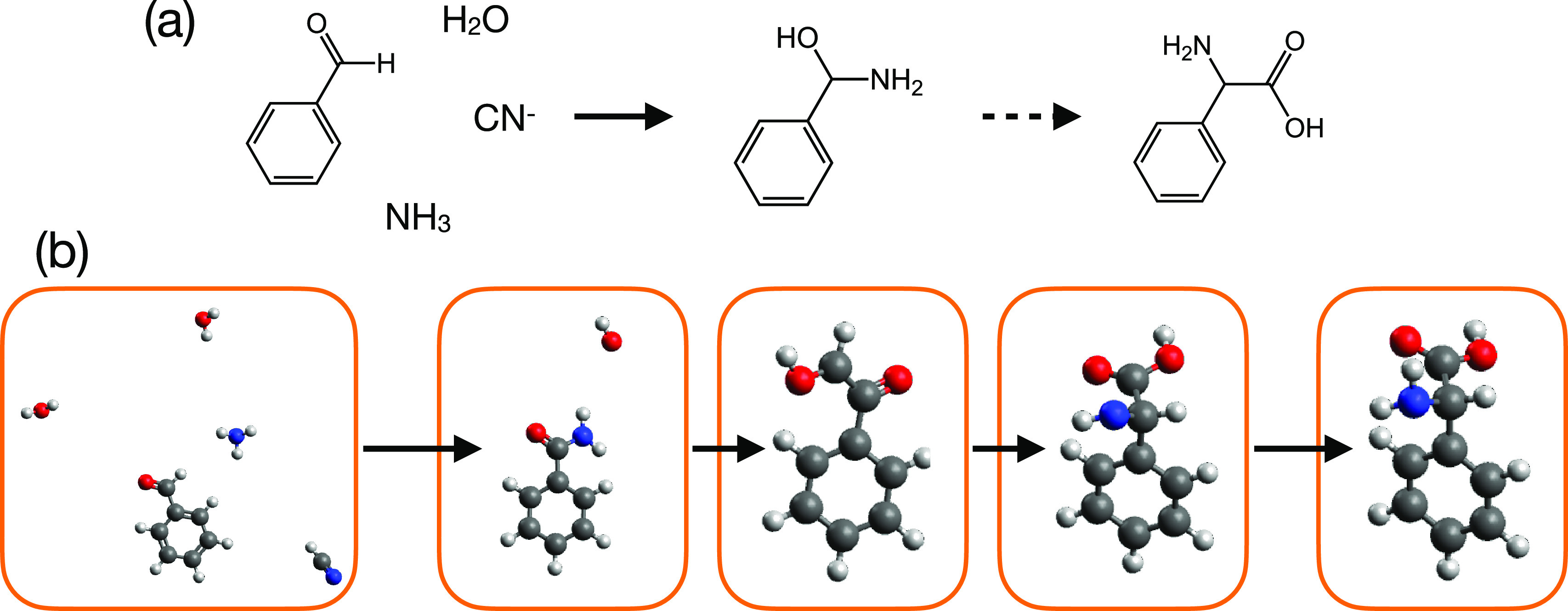
(a) Overview of Strecker reaction of benzaldehyde, yielding the related non-natural amino acid structure. (b) Representative DEGDS simulation of the same reaction; although DEGDS can readily identify reactions leading from reactants to products, it is often found that reactions are “out of sequence” in the overall mechanism, or nonrealistic intermediates, such as OH, are generated.

First, recent work has begun to explore the challenges associated with ARD in the context of multicomponent reactions (MCRs).^115−117^ MCRs enable construction of complex organic molecular structures through assembly of several molecular components, typically in a “cascade” or “domino” sequence of reactions wherein newly generated products at each step enable new reactivity in subsequent steps. MCRs are an increasingly fruitful route toward “green” chemical syntheses of complex organic molecules, offering high atom-efficiency and “one-pot” strategies that are desirable in organic synthesis.^118^ Our own interest in MCRs stems primarily from the methodological ARD challenges they afford, as illustrated in Figure 8; here, we show a representative DEGDS simulation for the Strecker synthesis (see, for example, ref (119)), a prototype MCR involving (in this example) reaction of benzaldehyde with ammonia, hydrogen cyanide and water. The mechanism of the Strecker synthesis is well-studied; the first “sequence” in the mechanism precedes with nucleophilic attack by NH_3_ followed elimination of water and reaction with a cyanide ion, yielding an intermediate aminonitrile species; in a subsequent sequence, protonation, subsequent nucleophilic attack by water, and elimination of ammonia yield the related (non-natural) amino acid. As such, the entire sequence of elementary steps in the Strecker reaction involves more than ten reaction steps, as well as multiple protonation/deprotonation reactions.

Under such conditions, using computational methods to identify appropriate mechanistic proposals is extremely challenging. First, the large number of independent reaction steps suggests that extensive CRN exploration is a necessity to ensure that all appropriate intermediates and reaction mechanisms are accessible. Second, methods based on stochastic selection of “next reactions”, such as SEGDS and DEGDS, struggle with these complex mechanisms due to the sheer number of possibilities for reactivity at each reaction step; specifically, although we have found that DEGDS can reliably generate *a* mechanism forming the target product, the stochastic selection of reactions and reactive atoms means that the majority of the proposed reaction steps involve reactions that are “out of sequence” in the overall mechanism. Finally, we note that multiple protonation/deprotonation steps, although common in organic reaction mechanisms, are nontrivial to account for. For example, a typical valence constraint in graph-driven methods is to enforce hydrogen atoms to have a valence of one, but this precludes generation of H^+^ during deprotonation steps, requiring instead a reaction partner to host the errant proton. This is, of course, chemically sensible (protons do not just walk off on their own in typical condensed-phase chemical reactions), but the demand to incorporate proton acceptors in the reactive system further increases complexity and hence increases the challenge of ARD. In summary, we suggest that MCRs, such as the Strecker reaction, stand as important challenges to computational ARD schemes; further developments in this area could have an impact on direct design of new MCRs in the important field of sustainable chemistry.

Second, as a different example of how graph-driven ARD schemes can be used to study kinetic systems, we highlight very recent work in our group aimed at using DEGDS to fold model proteins. For a given *known* protein structures, one can calculate an adjacency matrix at the level of amino acid residues; as a result, the same DEGDS methodology which has been applied to different chemical reaction systems can similarly be applied to generate protein-folding pathways, starting from random-coil structures and ultimately leading to the target folded structure. Initial work in this domain is promising, demonstrating that DEGDS can readily generate protein-folding walks in adjacency-graph space; further work is now underway to validate the DEGDS-generated paths for a variety of different proteins.

## Challenges to Reaction-Discovery Simulations

The examples above demonstrate what is currently possible using ARD methods based on graph-based strategies; given the impressive strides taken, in both reaction discovery and related methods such as MEP and TS finding, it should be clear that an enormous breadth of “chemical questions” are now accessible by such methods, spanning from interrogation of gas-phase reaction mechanisms in interstellar and combustion settings, to detailed study of catalytic cycles in homogeneous and heterogeneous systems.

However, large challenges remain in further popularizing the application and high-throughput automation of reaction-discovery schemes; in the following, we give a personal outline of current challenges that are particularly relevant to reaction networks for complex molecular systems.

### The Accuracy Problem

While enumerating possible reactants and products in complex CRNs is all well and good, the ultimate goals of gaining chemical insight or experimental rationalization can only be achieved when combined with characterization of the thermodynamic and kinetic parameters of each elementary reaction. This typically requires *ab initio* or semiempirical calculations, particularly geometry optimization (of reactants and products), TS finding, and free-energy evaluations.

While commonly used PES methods, such as DFT and DFTB, can often give good representations of the molecular structures of reaction endpoints, as well as TS geometries, a key problem in connecting quantitative CRN simulations to experimental studies lies in the requisite accuracy of energy evaluations. Accurate energy evaluations are particularly important when predicting the relative energies of TSs and reactants/products. If one adopts standard TST to calculate the reaction rates, then the relationship between the rate and the activation energy for the reaction, Δ*G*^⧧^, is
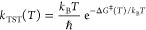
7where *T* is the temperature. If the “exact” activation energy, Δ*G*_e_^⧧^(*T*), was known, then the relative reaction rate predicted by some *ab initio* calculation method giving rise to the activation energy Δ*G*_c_^⧧^(*T*) is
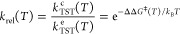
8where ΔΔ*G*^⧧^ = Δ*G*^c^(*T*) – Δ*G*^e^(*T*) is the difference between the calculated and “exact” activation energies. This exponential dependence of the reaction rate (and the relative rates) on the activation energy can lead to large errors in predicted rates for elementary steps in CRNs. For example, at *T* = 300 K, an error of 5 kJ mol^–1^ in an *ab initio* calculated activation energy would lead to a relative rate of either 0.13 or 7.46, depending on whether the calculated activation energy is under- or overestimated relative to the “correct” activation energy; as a result, subsequent kinetics modeling based on such rates could lead to quite different time scales compared to experimental observations.

This simple argument illustrates the clear challenge of *accurately* modeling reaction rates in CRNs. As is well-known, depending on calculation type, errors in DFT-based activation energies may be up to several tens of kJ mol^–1^; even the most accurate *ab initio* methods can have residual errors of a few kJ mol^–1^. As such, the explicit time scales associated with *ab initio* generated CRNs need to be carefully considered.

**Figure 9:**
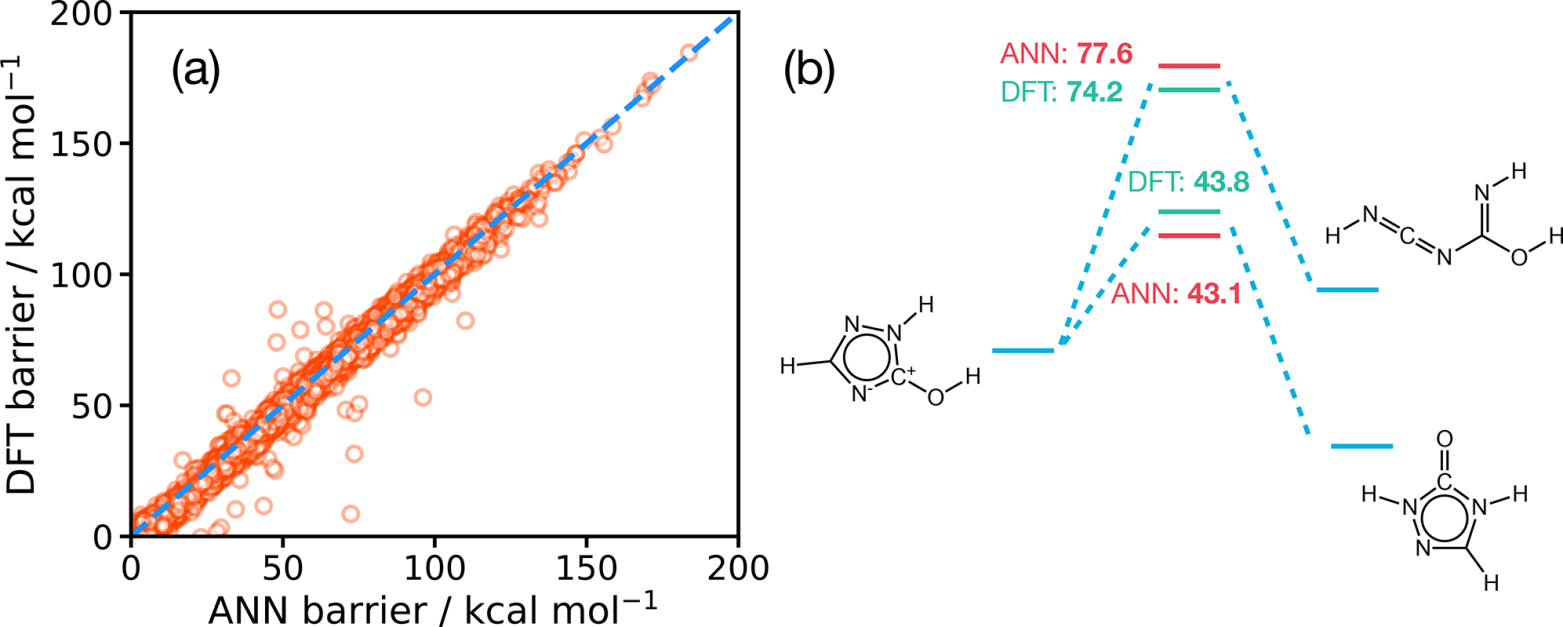
(a) Correlation plot showing ANN-predicted and actual DFT calculated barriers for a test set of around 6500 reactions; the ANN illustrated here was trained in the same way as described recently.^121^ (b) ANN prediction performance, compared to DFT activation energies, for two reactions (starting from the same reactants but leading to different products). Energies are given in kcal mol^–1^.

In addressing this accuracy challenge, the obvious solution is to use increasingly accurate *ab initio* calculation approaches, for example, moving up the “Jacob’s ladder” of DFT functionals.^120^ However, as noted below, computer-generated CRNs can rapidly become very large, with significant numbers of molecular species and reactions that must be characterized; in such cases, using high-accuracy *ab initio* methods for analysis of all species and reactions is not currently possible. An attractive alternative that has rapidly emerged over recent years is to use artificial-intelligence/machine-learning (AI/ML) strategies.^94,121−131^ For example, a number of different groups, including ours, have shown that, given sufficient data, one can train models such as artificial neural networks (ANNs) or Gaussian process regression (GPR) to accurately predict activation energies for elementary chemical reactions *given as input only the reactant and product structures* (hence circumventing accurate characterization of the TS). Typically, these AI/ML schemes use molecular descriptors for the reactants/products based on structural connectivity, most commonly the extended-connectivity (or Morgan) fingerprints;^132^ of course, as in many AI/ML applications, a range of different strategies have also been investigated. As representative performance levels, it is found that AI/ML schemes for activation energy prediction can achieve root-mean-square errors of 3 kcal mol^–1^ (i.e., 12.6 kJ mol^–1^) or less when compared to the reference (typically DFT) *ab initio* training data;^123,125,129,130,133^ this level of performance is typical of what can be achieved using curated organic chemistry data sets, such as that reported by Grambow et al., containing 10^4^ reaction examples or more.^122^ This is illustrated in Figure 9, which illustrates the predictive performance of an ANN trained to predict activation energies using the Grambow data set,^122^ with the descriptors for each reaction taking the form of Morgan *difference* fingerprints (i.e., the change in Morgan fingerprint upon moving from reactants to products) plus additional information about the energy change of reaction (typically calculated at DFT level). The RMSE prediction error for such a model is 3.8 kcal mol^–1^ (i.e., 15.9 kJ mol^–1^), which is comparable to previous work in this field; this achievable level of accuracy is also illustrated for two reaction examples in Figure 9. As also shown in recent work,^121^ this level of accuracy can be sufficient to provide a qualitative picture of CRN kinetics, but care must be taken in validating results and assessing quantitative predictions.

However, while these simulations demonstrate that AI/ML can accurately capture structural reactivity trends in activation energies, it is worth noting that DFT energies are typically used as the target training data here, meaning that the AI/ML naturally inherits the underlying inaccuracies of the *ab initio* approach employed. This suggests that there is a future opportunity to develop new AI/ML schemes which minimize the number of training examples required to achieve a high degree of accuracy; for example, if an accurate AI/ML could be trained using just a couple of thousand reactions, then much higher accuracy *ab initio* schemes could be used to generate the requisite training data. The development of such low-data AI/ML methods is an active area of research and will surely transfer to the domain of CRN prediction in the coming years. In addition, the incorporation of known *experimental* data, such as reaction rates or formation enthalpies, into data sets for AI/ML training could have a similarly important impact, provided that the challenges of training using mixed-origin data can be adequately addressed.

**Figure 10:**
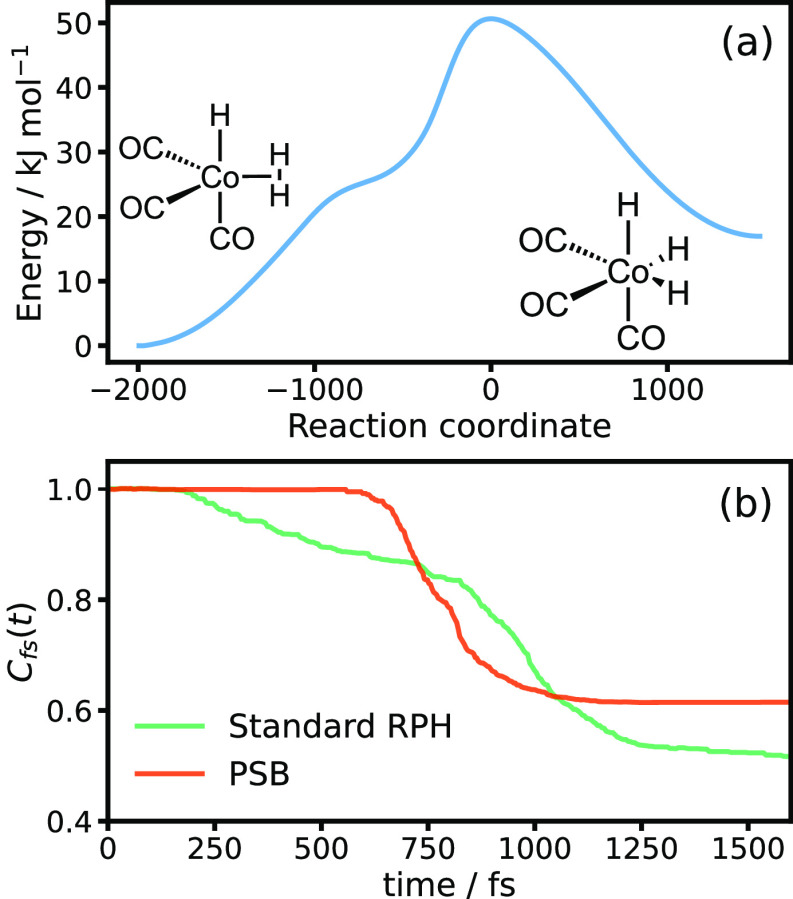
(a) MEP for the insertion of molecular hydrogen H_2_ at the cobalt center of HCo(CO)_3_, the active catalytic species in the Heck–Breslow hydroformylation previously studied by ARD simulations. (b) Calculated flux-side correlation functions given by a standard RPH simulation (requiring multiple Hessian matrix evaluations along the MEP) and by our recent work in which Hessian propagation schemes (in this case, Powell–symmetric–Broyden [PSB]) are used to build the RPH.^135^

As a final point, it is worth noting that inaccuracies in activation energies are not the only source of error in CRN characterization. In particular, the true reaction rate for a given elementary reaction can be written as^134^
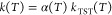
9where α(*T*) is the temperature-dependent transmission coefficient, which corrects the TST rate *k*_TST_(*T*) for the influence of dynamical recrossing events. As such, the standard TST assumption that α(*T*) = 1 can itself introduce a significant error in those cases when TS recrossing effects (such as that caused by significant solvent interactions) are large. Correcting for such effects demands evaluation of α(*T*); this is in itself a computationally expensive exercise, requiring thermal averaging of a number of MD trajectories (typically on an *ab initio* PES or reactive force-field model) initiated at the TS in order to evaluate the flux-side correlation function. To address this inefficiency, we have recently shown^135^ how α(*T*) can be accurately and efficiently approximated using a reaction-path Hamiltonian (RPH)^136^ model parametrized using information available from standard NEB optimization of the MEP; importantly, we have also demonstrated that RPH construction can be further accelerated by using a variety of Hessian propagation schemes, thereby avoiding expensive *ab initio* Hessian calculations for a dense set of intermediate images.^137,138^ As shown in Figure 10 for the example reaction of molecular hydrogen association at the cobalt center in HCo(CO)_3_, relatively simple Hessian update schemes combined with MD simulations using the RPH model enable accurate approximation of α(*T*), even for reactions in which recrossing is quite significant. Such methods demonstrate how one can improve on the treatment of TST rate theory in a simple computational scheme; although we note that the challenge of accurately modeling the underlying PES remains. Finally, it is worth noting that anharmonic models for calculating molecular free energies have also been developed and tested, thereby reducing errors introduced by treating molecules as harmonically oscillating rigid rotors; work in this field remains active but yet again is tempered by the demands for PES accuracy.^139,140^

### The Transition-State Problem

Related to the challenge of accurate modeling of reactions is the problem of TS location; this is an important prerequisite for TST and transmission-coefficient evaluations on the road to accurate reaction rates. Given the central role the TS plays in reactions, an enormous number of different TS-finding schemes have been developed over the years, including (but not limited to) the synchronous transit method^141^ and eigenvector-following schemes.^142^ In many standard applications, these approaches can work well; however, in the setting of CRN generation, where one could potentially be generating hundreds or thousands of unique elementary chemical reactions, TS-finding can present challenges to high-throughput automation. For example, TS-finding algorithms can be quite sensitive to the initial configuration; as such, preconditioning schemes can be useful, aiming to preferentially orientate molecules in space before TS-finding begins.^69^ Furthermore, give numerical noise in typical self-consistent-field-type calculations, accurately converging TS geometries to unambiguously identify single negative eigenvalues is often challenging too.

As such, TS-finding on the scale demanded for *automated* CRN generation still requires some development to provide robust strategies for restarting TS searches with intelligent initial configurations (perhaps generated by ML schemes trained using examples of identified TS structures); in addition, in light of the discussion on accuracy above, it is clear that TS-finders that minimize the required number of force and Hessian matrix evaluations will remain in demand as computationally accurate energy evaluation schemes are increasingly employed. Finally, it is worth noting that conformational flexibility in reactant species must also be accounted for, especially for more complex molecular systems and where different energetically accessible conformers might reasonably be expected to have quite different reactivity.

### The Search-Space Problem

Put simply, the chemical reaction space explored during CRN generation can be enormous. In the simplest case of an *N*-atom reactive system, an upper limit to the number of different chemical species that can be generated is 2^*N*(*N*–1)/2^, accounting for all possible bond arrangements and ignoring the distinction between single and multiple bonds. The number of “sensible” (or physically realizable) available structures will certainly be less than this upper bound but may still also be an enormous number of different molecular species.

In any case, the vast growth in the size of chemical space as the complexity and size of a “virtual reaction vessel” increases places significant demands on computational chemistry. As noted above, high-throughput, automated workflows (merging reaction discovery, TS finding, and quantum chemical calculations) are increasingly being used to address such challenges, although even these workflows will eventually buckle under the challenge of accurately characterizing large CRNs. Furthermore, such automated high-throughput CRN generation comes at enormous computational expense, as well as real-world energy-consumption costs that should not be overlooked.

As noted above, AI/ML schemes potentially offer new opportunities to address challenges associated with the size of chemical space in CRNs. Rapid evaluation of reaction thermodynamics and kinetics using trained ML models can clearly accelerate CRN generation; a number of examples of this strategy have now been reported, as noted above. More broadly, however, AI could offer a way to *intelligently* explore chemical reaction space starting from a given set of reactant species. Here, for example, using probabilistic models that capture the same sort of rational understanding of functional groups and common reaction classes that is embodied in the typical organic synthesis expert, an AI could aim to predict the “most physically plausible” set of onward reactions, rather than the more brute-force CRN generation that is characterized by many graph-based approaches at present. This incorporation of “chemical common sense” is already appearing in many ARD schemes, for example, in the form of bond and atomic valence constraints being used to limit formation of unusual molecular species; integration of AI/ML, trained on large computational and/or experimental reactive databases, could further boost this strategy.

### The “Stamp-Collecting” Problem

As demonstrated here, using efficient algorithms for chemical space exploration, combined with the sheer computing power and storage capacity available to typical computational chemists, we are quickly moving into a position in which we can generate *enormous* CRNs for complex and diverse sets of reactant molecules. CRNs containing many thousands (or more) of species and reactions could quickly become quite standard, providing detailed reaction models of a variety of different chemical processes.

Two important questions are “When should we stop? How do we know when our autogenerated CRNs are satisfactorily complete and accurate that we can sufficiently answer physical questions posed?”. This may be considered an obsolete question, given that computational chemists typically have access to enormous computational *storage* resources, but it is worth bearing in mind when starting CRN generation that relentless reaction sampling might be wasting valuable resources which could be used in a more focused fashion, as we have already noted above in regard to the energy cost of computing time.

This idea, seeking to avoid simply “stamp-collecting” chemical reactions, itself presents opportunities. For example, perhaps a centralized curated database of previously generated reactions and/or reaction templates could help constant repetition in generating and characterizing already known reactions; a “Google maps” for reactive chemistry, generated by *ab initio* quantum chemistry, would provide a valuable resource for CRNs and AI/ML methods alike. Furthermore, in the age of open data, enabling free, perpetual access to such a resource could have further transformative impacts for science as a whole. From this viewpoint, as noted in this article, it seems that we increasingly have access to the computational tools required to generate such a road map of chemical reactivity “from the ground up”.

## Conclusions

In this article, we have highlighted a series of projects aimed at developing and investigating new simulation methods to study complex reactive systems; in particular, we have focused on simulation strategies based on the concept of bonding graphs. These mathematical structures form a useful starting point for a number of algorithms developed over the last couple of decades; however, the growth of AI/ML methods, in addition to increasingly inexpensive high-performance computing hardware to enable *ab initio* electronic structure calculations, mean that new opportunities for ARD methods have rapidly advanced in the past decade or so. Such ARD schemes are now increasingly available to study complex chemical reactions; addressing some of the challenges posed here could further boost this research field. In the long term, as the interaction between theory and experiment (through concepts of CRNs) is strengthened, one can envisage the growth of “digital twins” of reactive chemical set-ups, providing integration of “real world” and “virtual” data; this could be a significant boost to design of molecular functional systems, such as new green catalysts. The central concept of CRNs, as well as the continued growth of computational ARD schemes, is surely increasingly set to drive this field forward.
